# The Impact of Lipid Types and Liposomal Formulations on Osteoblast Adiposity and Mineralization

**DOI:** 10.3390/molecules23010095

**Published:** 2018-01-02

**Authors:** Shun-Fu Chang, Chih-Chang Yeh, Pin-Jyun Chen, Hsin-I Chang

**Affiliations:** 1 Department of Medical Research and Development, Chang Gung Memorial Hospital Chiayi Branch, Chiayi 61363, Taiwan; sfchang@cgmh.org.tw; 2 Department of Orthopaedics, Chiayi Branch, Taichung Veterans General Hospital, No. 600, Sec. 2, Shixian Road, West District, Chiayi City 60090, Taiwan; yeh215kimo@yahoo.com.tw; 3 Department of Biochemical Science and Technology, National Chia Yi University, No. 300, Syuefu Rd, Chiayi City 60004, Taiwan; linda041134@gmail.com

**Keywords:** lipid, liposomal formulation, osteogenesis, lipid droplet accumulation, osteoblast

## Abstract

Recent studies have demonstrated that fat accumulation in bone cells is detrimental to bone mass. Both adipocytes and osteoblasts are derived from common multipotent mesenchymal stem cells (MSCs) and hence the presence of fat may increase adipocyte proliferation, differentiation and fat accumulation while inhibiting osteoblast differentiation and bone formation. Lipids are common constituents in supramolecular vesicles (e.g., micelles or liposomes) that serve as drug delivery systems. Liposomal formulations such as Meriva^®^ were proven to decrease joint pain and improve joint function in osteoarthritis (OA) patients. In this study, we evaluated how lipid types and liposomal formulations affect osteoblast behavior including cell viability, differentiation, mineralization and inflammation. Various liposomal formulations were prepared using different types of lipids, including phosphatidylcholine (PC), 1,2-dioleoyl-sn-glycero-3-phospho-ethanolamine (DOPE), cholesterol (Chol), 3β-[*N*-(*N*′,*N*′-dimethylaminoethane)-carbamoyl] cholesterol hydrochloride (DC-cholesterol HCl), and 1,2-dioleoyl-3-trimethylammonium-propane chloride salt (DOTAP) to investigate the impact on osteoblast differentiation and inflammation. The results indicated that cationic lipids, DC-cholesterol and DOTAP, presented higher dose-dependent cytotoxicity and caused high level of inflammatory responses. Due to the natural properties of lipids, all the lipids can induce lipid droplet formation in osteoblasts but the level of lipid droplet accumulation was different. In comparison with cationic lipids, neutral lipids induced less adiposity, and maintained high osteoblast mineralization. Similar to previous researches, we also confirmed an inverse relationship between lipid droplet formation and osteoblast mineralization in 7F2 mouse osteoblasts. Importantly, PC containing liposomes (PC only and PC/DOTAP) suppressed IL-1β-induced gene expression of COX-2 and MMP-3 but not Chol/DOTAP liposomes or DC-Chol/DOPE liposomes. Taken together, we suggested that PC contained liposomes could provide the best liposomal formulation for the treatment of bone diseases.

## 1. Introduction

Osteoblast progenitors have the potential to differentiate into osteoblasts or adipocytes. The commitment and differentiation of osteoblast progenitors towards an adipogenic or osteogenic cell fate depend on a variety of signaling and transcription factors. Increased evidence suggests that an inverse correlation exists between adipogenesis and osteogenesis in mesenchymal stem cells (MSCs) [1]. Previous studies have demonstrated that the disruption of the balance between osteogenesis and adipogenesis could lead to bone diseases such as osteoarthritis (OA) and osteoporosis [2]. Van de Vyver et al. mentioned that chronic administration of the insulin-sensitising drugs, thiazolidinediones, results in low bone mineral density and fatty bones because MSC may differentiate towards adipogenesis rather than osteogenesis [3]. Thus, balanced osteoblastic and adipogenic differentiation is critical for the maintenance of healthy bone and lean body composition [4].

The presence of fat may increase adipocyte proliferation, differentiation and lipid droplet accumulation associated with a decrease in osteoblast differentiation and bone formation. In the clinical study, more adipogenesis than osteogenesis in marrow is deleterious to bone as the symptoms like osteoporosis, diabetes mellitus, and OA [5]. However, most of the interest has focused on the potential of human MSCs to differentiate into osteoblasts or adipocytes. The role of adiposity in osteoblasts or osteoblast progenitors is still unclear. Our previous study found that liposome treatment can suppress osteoblast mineralization through the lipid droplet formation [6]. Therefore, we aim to further investigate the impact of lipid types and liposomal formulations on lipogenesis, osteogenesis and inflammation responses in 7F2 osteoblasts.

OA is a progressive degenerative joint disease, characterized by the breakdown of joint cartilage. Several risk factors for OA have been previously identified, including genetic predisposition, mechanical stress, obesity, bone deformities, previous injury, and aging. There is a growing evidence indicating that the progression of OA is correlated with an upregulation of inflammatory processes [7]. The most common symptoms of osteoarthritis are joint pain, stiffness, swelling and bone deforming. There are a number of treatments are available to relieve the symptoms such as lifestyle modifications (exercise or diet controlling), medication (glucosamine supplement or hyaluronic acid injection), physical therapy and surgery. Liposomal formulation is a new medication for osteoarthritis treatment. Previous studies demonstrated that lipid drug carriers such as Meriva can reduce inflammatory response, joint swelling, and bone erosion in the patients with osteoarthritis [8]. Hence, the anti-inflammatory activities of various liposomal formulations were measured in 7F2 osteoblasts for inflammatory bone disorders.

Lipids are common constituents of various supramolecular vesicles (e.g., micelles or liposomes) that serve as drug delivery systems. In this study, we selected 5 different types of lipids including phosphatidylcholine (PC), 1,2-dioleoyl-sn-glycero-3-phosphoethanolamine (DOPE), cholesterol (Chol), 3β-[*N*-(*N*′,*N*′-dimethylaminoethane)-carbamoyl] cholesterol hydrochloride (DC-Cholesterol), and 1,2-dioleoyl-3-trimethylammonium-propane chloride salt (DOTAP), which have been applied for liposomal formulations in clinical use, to investigate the effect on lipid droplet formation and mineralization in 7F2 osteoblasts. PC, DOPE and DOTAP are the model of long chain lipids. Additionally, PC and DOPE are zwitterionic neutral lipid whereas DOTAP is a cationic lipid. It is worth noting that DOPE is a helper lipid in cationic liposomes because the incursion of DOPE into cationic liposomes can facilitate membrane fusion and exhibit low cytotoxicity and good transfection efficiency [9]. Similar to DOPE, the incursion of cholesterol into the liposomal formulations can increase liposome membrane rigidity and that may improve in vivo and in vitro stability of the liposomes [10]. Besides, a cationic cholesterol derivative, DC-Cholesterol was commercially available liposome reagent and DC-Chol/DOPE cationic liposomes have been successfully used as a vector in clinical trials for treating melanoma and cystic fibrosis [11]. Therefore, the first objective of this study is to evaluate the potential of lipids (PC, DOPE, Chol, DC-Chol, and DOTAP) as liposomal delivery system in the treatment of bone diseases.

Liposome is an artificially-prepared spherical vesicle composed of a lamellar phase phospholipid bilayer. The researches have showed that the increase of liposome stability in vitro and in vivo is based on lipid compositions and ratio [12]. Generally, the main components of liposomes are phospholipids such as PC and DOPE but some are incorporated with Chol [13,14,15]. PC-containing liposomes previously were found to have the highest incorporation of drug in comparison with other lipids [16]. DOTAP, a double chain monovalent quaternary ammonium lipid, is widely used as an in vitro transfection agent by forming cationic liposomes for gene delivery [17]. In the study reported by Baczynska et al., PC/DOTAP showed a significant high liposome uptake efficiency in human colon cancer cells (CX-1.1) compared to that of liposomes containing pure egg-PC [16]. Moreover, the incorporation of Chol into DOTAP liposomes can improve transfection efficiency through the decrease of the membrane fluidity, the reduction of particle charge density and improving resistance of liposome carriers to aggregation [18]. On the other hand, DC-Chol/DOPE liposomes also showed good efficient transfection in gene delivery and hence DC-Chol/DOPE liposomes have been classified as one of the most efficient gene delivery systems [19]. Goyal and Huang reported that DC-Chol/DOPE cationic liposomes have several attractive characters such as low cytotoxicity, less immunogenicity, ease of utilization and high stability [11]. In this study, we formulated PC, PC/DOTAP, Chol/DOTAP and DC-CHol/DOPE liposomes to investigate their impacts in osteoblast behavior including viability, differentiation, mineralization and inflammation.

## 2. Results

### 2.1. The Effect of Lipid Types on Cell Viability of 7F2 Osteoblasts

In order to investigate the cytotoxicity of lipids on 7F2 mouase osteoblasts, the cells were incubated with the increasing concentrations of lipids (PC, DOPE, Chol, DC-Chol, and DOTAP) from 0 to 20 μg/mL for 24 h and measured by MTT assay. Due to poor aqueous solubility, all the lipids were dissolved in ethanol (EtOH) for experimental studies. Ethanol is edible and have been used as the solvent for liposome preparation.

**Figure 1:**
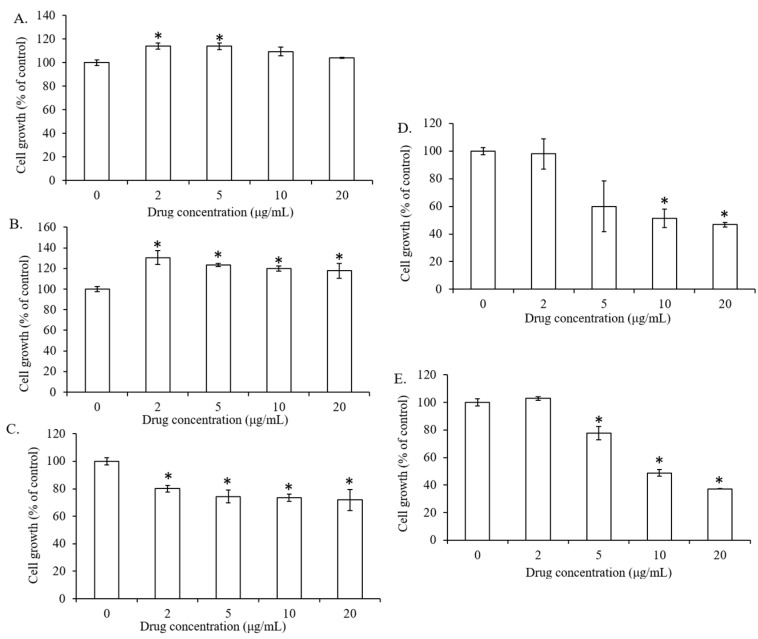
The effect of different concentrations of lipids on osteoblast growth. 7F2 osteoblasts were incubated with different concentrations of lipids ((**A**) PC; (**B**) DOPE; (**C**) Chol; (**D**) DC-CHol; (**E**) DOTAP) for 24 h and then examined by MTT assay. Data are expressed by the mean of percent cell viability compared to control after exposured for 24 h ± standard deviation (*n* = 3–6). * *p* < 0.01, relative to the untreated (0 μg/mL) cells.

Akopian et al. and Kim et al. also used ethanol as solvent for cholesterol, phosphatidylcholine and polyene phosphatidylcholine to evaluate their effects in vitro and in vivo [20,21]. PC and DOPE showed no cytotoxicity in 7F2 osteoblasts and cell number was slightly increased (Figure 1A,B), while Chol, DC-Cholesterol and DOTAP exhibited dose-dependent cytotoxicity (Figure 1C–E). Similar to DC-Chol (Figure 1D), the cytotoxicity activity of DOTAP started at the concentration of 5 μg/mL (less than 80% cell viability, Figure 1E). Therefore, we selected 2 μg/mL as the lipid concentration for the following experiments.

### 2.2. The Influence of Lipid Types on the Lipid Droplet Formation of 7F2 Osteoblasts

**Figure 2:**
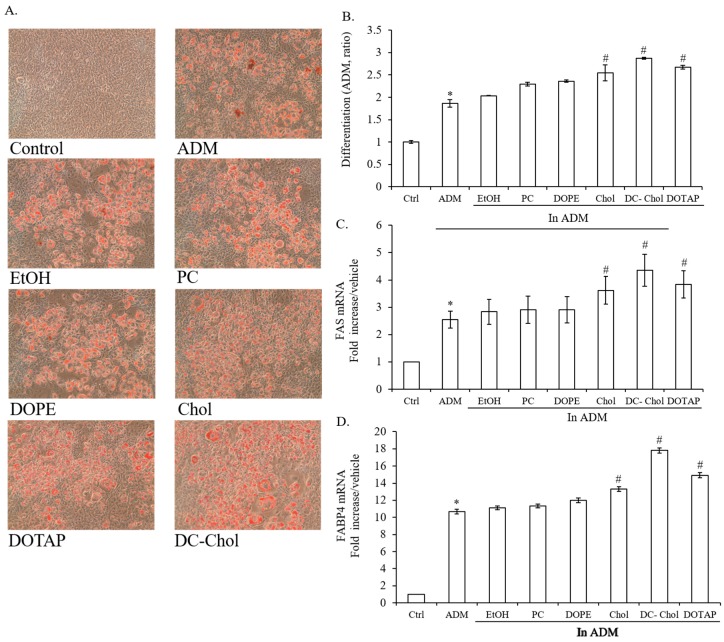
The influence of lipid content on osteoblast adipogenesis. (**A**) Histochemical staining of lipid accumulation. 7F2 osteoblasts were treated with various lipids in the presence of ADM for 7 days to induce adipogenesis and visualized by Oil Red O Staining (×100 magnification, *n* = 3). Photographs show representative adipocyte cultures under bright field. The red spot represents oil droplet stained by Oil Red O; (**B**) The quantitation of lipid accumulation; The red staining was extracted with 100% isopropanol and quantified as described in Materials and methods; (**C**,**D**) The expression of adipogenic markers, FAS and FABP4. The mRNA expressions of FAS and FABP4 were measured by real-time PCR analysis after 7 days incubation with ADM. The data are presented as the means ± standard deviation. * *p* < 0.01, relative to control, ^#^ *p* < 0.01, relative to EtOH-treated cells.

Although lipid droplet accumulation has been indicated in many cell types such as 3T3 fibroblasts, hepatic stellate cells and smooth muscle cells, there has been little evidence that osteoblasts possess this characteristic [21,22,23]. In here, 7F2 osteoblasts were incubated with adipogenic differentiation medium (ADM) for 7 days to induce adipogenesis. Representative bright field images demonstrated that ADM stimulated massive lipid droplet formation in 7F2 osteoblasts as indicated by Oil Red O staining (Figure 2A).

In contrast to ethanol treated cells, lipids stimulated more lipid droplet accumulation in 7F2 osteoblasts, particularly Chol, DC-Chol, and DOTAP. Moreover, cationic lipids, DC-Chol and DOTAP induced more lipid accumulation in 7F2 osteoblasts than neutral lipids, Chol and PC (*p* < 0.01, Figure 2B). *Fatty acid synthase* (FAS) is a multi-enzyme protein that catalyzes the formation of long-chain fatty acids from acetyl-CoA, malonyl-CoA and NADPH and has been used as the adipogenic marker [24]. The fatty acid binding protein 4 (FABP4), commonly known as adipocyte protein 2 (aP2), has been extensively used as a marker for differentiated adipocytes. FABP4 is a carrier protein for fatty acids that is primarily expressed in adipocytes and macrophages [25]. 7F2 osteoblasts were cultured with different types of lipids in the presence of ADM for 7 days and operated by real-time RT-PCR to determine the impact of lipid content on the gene expressions of FAS and FABP4. Compared with control, ADM treatment individually induced 2.5 and 10 fold higher expressions of FAS and FABP4 (Figure 2C,D). In addition, lipids such as Chol, DC-Chol, and DOTAP stimulated higher expression level of FAS and FABP4 and hence Chol, DC-Chol, and DOTAP had the abilities to promote lipogenesis in 7F2 osteoblasts, while PC and DOPE showed less impact on lipid droplet formation.

### 2.3. The Impact of Lipid Types on Osteogenesis in 7F2 Osteoblasts

**Figure 3:**
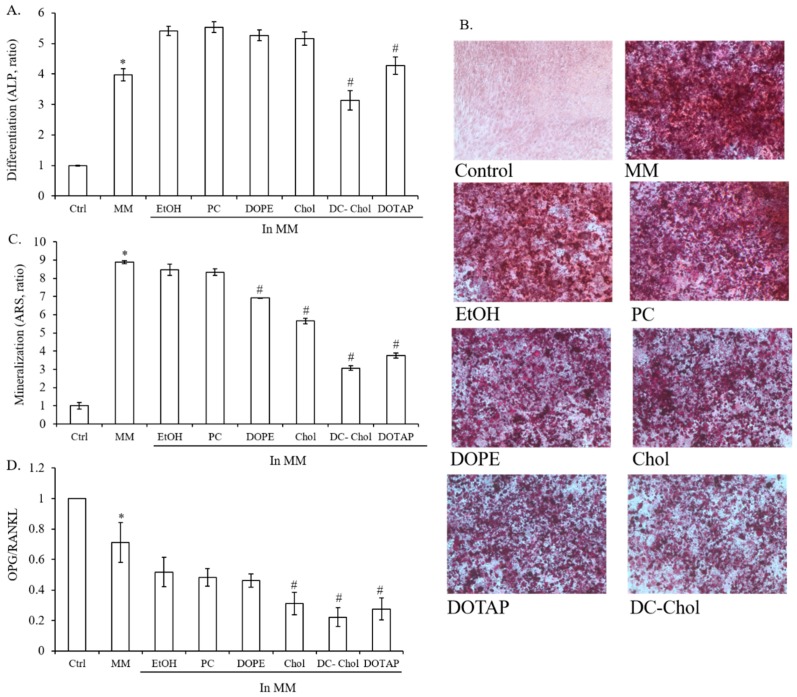
The impact of lipid types on osteoblasts differentiation and mineralization. 7F2 osteoblasts were treated with MM (50 μg/mL ascorbic acid and 5 mM β-glycerophosphate) to induce osteoblast differentiation and mineralization. (**A**) The ALP activity of 7F2 osteoblasts in the absence and presence of MM with various types of lipids; (**B**) Histochemical staining of mineralization deposition. 7F2 osteoblasts were cultured in absence or presence of MM with different types of lipids for 14 days and calcium ion deposition was visualized by Alizarin Red S staining (×100 magnification, *n* = 3). Red staining represents mineral deposition. Photographs represent osteoblast cultures under bright field; (**C**) The quantification of osteoblast mineralization. Alizarin Red S was extracted with 10% cetylpyridinium chloride and quantified as described in Materials and methods; (**D**) The effect of lipid types on the OPG/RANKL ratio of 7F2 osteoblasts. Cells were incubated in the absence or presence of MM with various types of lipids for 3 days and analyzed by real-time RT-PCR to investigate gene expression of OPG and RANKL (relative to GADPH). The data are presented as the means ± S.E. * *p* < 0.01, relative to control, ^#^ *p* < 0.01, relative to EtOH-treated cells.

In order to investigate the potential of lipid types on osteogenesis, 7F2 osteoblasts were cultured with mineralization medium (MM) for 10 days to measure alkaline phosphatase (ALP) activities. Under MM condition, ALP activity was around 4 times higher than control. In comparison with ethanol treated cells, cationic lipids (DC-Chol and DOTAP) reduced the level of ALP activities but not PC, DOPE, and Chol (Figure 3A). To further study the impact of lipid types on 7F2 osteoblast mineralization, Alizarin Red S (ARS) staining was used to evaluate calcium deposits after 14 days of incubation with MM which contained 50 μg/mL ascorbic acid and 5 mM β-glycerophosphate (Figure 3B). Incubation with MM could stimulate the mineralization level of 7F2 osteoblasts around 9 times higher than control. In contrast to ethanol treated cells, most types of lipids suppressed osteoblast mineralization significantly in 7F2 cells, except PC. Moreover, DC-Chol and DOTAP demonstrated 54% and 45% reductions of osteoblast mineralization to the cells treated with Chol and PC, respectively. As expected, the impact of lipids on osteogenesis is opposite to lipogenesis in 7F2 osteoblasts on matter with what kind of lipids. Similar to previous results, neutral lipids, especially PC, maintained better osteoblast mineralization than cationic lipids (Figure 3C). Receptor activator of nuclear factor κβ ligand (RANKL), a member of tumor necrosis factor (TNF) family, can activate nuclear factor-κβ (NF-κβ) through RANK-RANKL specific binding to stimulate the survival, differentiation and activation of osteoclasts [26]. Because osteoprotegerin (OPG) interfere the interaction between RANKL and RANK and hence the ratio of OPG/RANKL is correlated with a significant inhibition of the resorptive activity [27]. Although MM and EtOH showed the lower level of OPG/RANKL ratio than control, the ratio of OPG/RANKL was decreased even more by Chol, DC-Chol and DOTAP. Our results suggest that lipids, particularly Chol, DC-Chol and DOTAP, may stimulate osteoclastogenesis through the inhibition of osteoblast mineralization and the suppression of OPG/RANKL ratio (Figure 3D).

### 2.4. The Impact of Lipid Types on the Inflammatory Response of IL-1β-stimulated 7F2 Osteoblasts

Towle et al. have detected IL-1α and Il-1β cytokines in the chondrocytes of mildly osteoarthritic human cartilage and which may play a role in the pathological joint destruction in osteoarthritis [28]. Cytokines such as IL-1 produced by activated synoviocytes, mononuclear cells or by articular cartilage itself significantly up-regulate the gene expressions of pro-inflammatory mediators, cyclooxygenase 2 (COX-2) and metalloproteinase (MMP)-3 [29]. In here, we used IL-1β to induce inflammation responses on 7F2 osteoblasts for investigate the impact of lipid types on the expressions of inflammatory cytokines.

**Figure 4:**
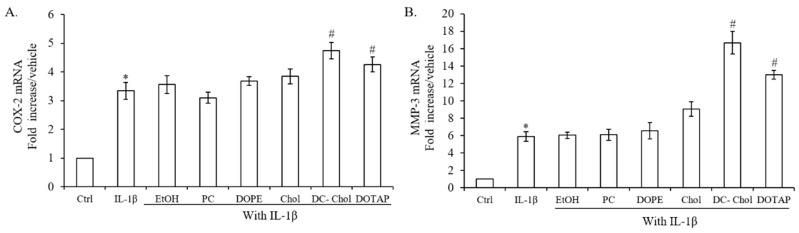
The effect of lipid types on the expression of COX-2 and MMP-3 of 7F2 osteoblasts in the presence or absence of 10 ng/mL IL-1β stimulation. (**A**,**B**) COX-2 and MMP-3 mRNA expressions of 7F2 osteoblasts. Real-time PCR analysis was operated in 7F2 osteoblasts after 4 h treatment of IL-1β. Gene expression level of COX-2 and MMP-3 demonstrated similar results. Chol, DC-Chol, and DOTAP amplified inflammatory responses on IL-1β-stimulated 7F2 osteoblasts, and DC-Chol showed the strongest inflammatory responses. On the other hand, PC exhibited the minor effect on inflammation of 7F2 osteoblasts. The data are presented as the means ± S.E. * *p* < 0.01, relative to control, ^#^ *p* < 0.01, relative to EtOH-treated cells.

The gene expressions of COX-2 and MMP-3 in IL-1β-stimulated 7F2 osteoblasts are around 3 and 6 fold higher than control (Figure 4A,B). In comparison with ethanol-treated cells, cationic lipids including DC-Chol, and DOTAP appeared to stimulate more gene expression of COX-2 and MMP-3 expressions in IL-1β-stimulated 7F2 osteoblasts but not neutral lipids, PC and DOPE. Therefore, cationic lipids may stimulate inflammatory responses in osteoblasts to cause ECM degradation and bone loss.

### 2.5. The Effect of Liposomal Formulations on Particle Size and Stability and Cell Viability of 7F2 Osteoblasts

**Figure 5:**
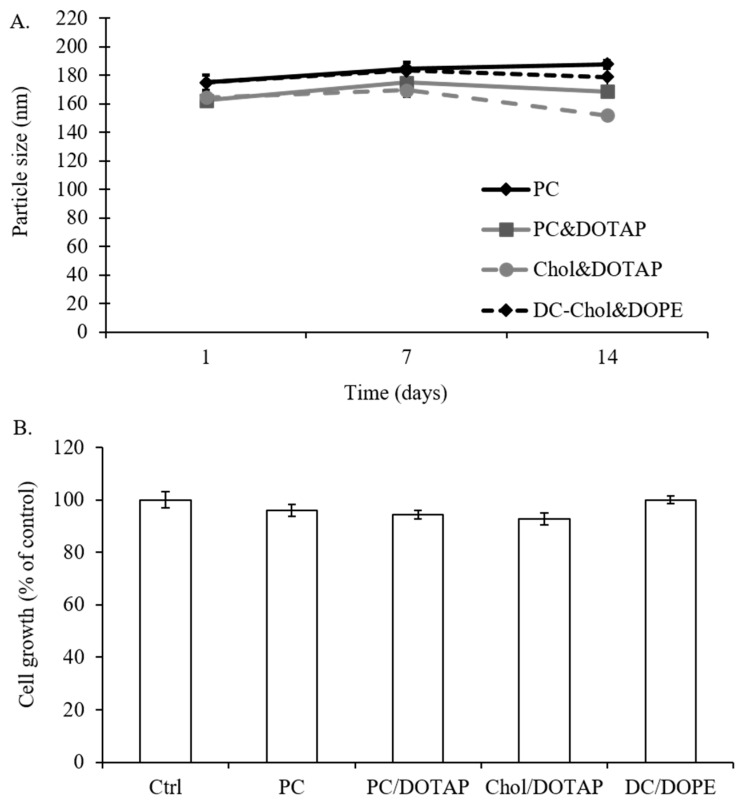
The effect of liposomal formulations on liposomal stability and 7F2 osteoblast viability. (**A**) The stability of liposomes prepared with different lipid compositions at 4 °C for 14 days; (**B**) The cell growth of 7F2 osteoblasts after 24 h treatment with 2.5 μg/mL liposomal formulations. Data are expressed by the mean of percent cell viability compared to control after exposured for 24 h ± standard deviation (*n* = 3–6).

PC, DOPE, Chol, DC-Chol, and DOTAP are common lipid components in liposomal formulations of clinically used products and ongoing clinical trials [11,13]. Based on the previous liposomal formulations, four types of liposomal formulations were prepared: (1) liposomes prepared with PC only (PC liposomes) (2) liposomes prepared by 1:1 ratio of PC and DOTAP (PC/DOTAP liposomes) (3) liposomes prepared by 1:1 ratio of cholesterol and DOTAP (Chol/DOTAP liposomes) (4) liposomes prepared by 1:1 ratio of DC-Cholesterol and DOPE (DC-Chol/DOPE liposomes) for the following experiments [6,16,30,31,32]. After rehydration, liposomes were characterized with a mean diameter greater than 2000 nm and a very large size distribution. In order to obtain liposomes with smaller and uniform size distributions, the lipid extrusion with polycarbonate filters was carried out. This procedure provided small multilamellar vesicles with mean diameters ranging from 160–180 nm. In our evaluation study for particle size, DOTAP were found to be a very useful reducing agent in liposomal formulation (PC/DOTAP liposomes and Chol/DOTAP liposomes), whereas PC liposomes and DC-Chol/DOPE liposomes tended to produce slightly larger particles (Table 1). Therefore, the presence of DOTAP in liposomal formulations may reduce the particle size but not DC-Chol. The mean size of a drug carrier is an important requisite that can influence the delivery device's biological effectiveness. In order to see the change of particle size of various liposomal formulations over time, a particle stability study of prepared liposomal suspensions was carried out at a temperature of 4 °C for a period of up to 2 weeks. At expected, all the liposomal formulations were stable for at least 2 weeks because there were less than l0% variation in particle size at day 14. (Figure 5A). Moreover, there was no significant cytotoxicity determined with all the liposomal formulations at the concentration of 2.5 μg/mL and which would be the concentration of liposomes used for the following experimental studies (Figure 5B).

### 2.6. The Impact of Liposomal Formulations on the Lipid Droplet Formation in 7F2 Osteoblasts

**Figure 6:**
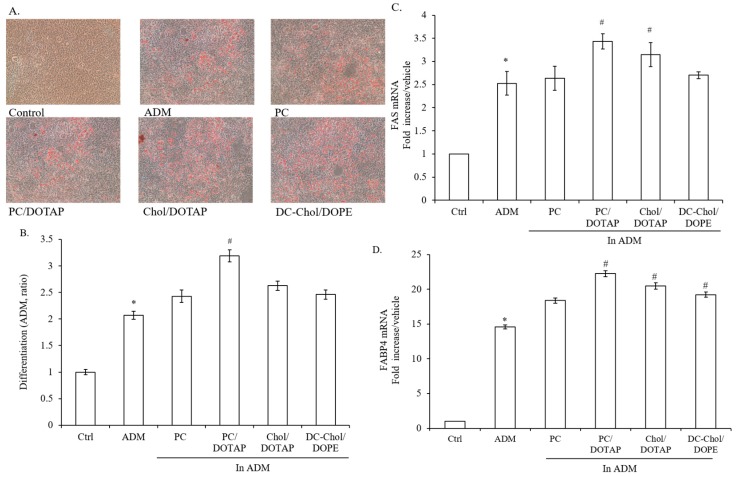
The influence of liposomal formulations on adipogenic differentiation of 7F2 osteoblasts. (**A**) Histochemical staining of lipid accumulation. 7F2 osteoblasts were visualized by Oil Red O Staining (×100 magnification, *n* = 3). The red spot is oil droplet stained by Oil Red O. Photographs show representative adipocyte cultures under bright field; (**B**) The quantitation of lipid accumulation; (**C**,**D**) The gene expressions of adipogenic markers, FAS and FABP4. The data are presented as the means ± S.E. * *p* < 0.01 relative to Control, ^#^ *p* < 0.01 relative to ADM-treated cells.

Similar to Figure 2A, Oil Red O staining was used to detect lipid accumulation in cells treated with various liposomal formulations (Figure 6A). The quantitation of Oil Red O staining exhibited that PC, Chol/DOTAP and DC-Chol/DOPE liposomes slightly increased lipid droplet formation in comparison with ADM-treated cells but not in statistical significance (Figure 6B)*.* Moreover, PC/DOTAP liposomes induced lipid accumulation in 7F2 osteoblasts 1.5 fold higher than ADM-treated cells. Next, we examined the influence of liposomal formulations on gene expressions of FAS and FABP4. PC/DOTAP liposomes had 1.4 and 1.5 fold increase in gene expressions of FAS and FABP4 in contrast to ADM-treated cells. In addition, Chol/DOTAP liposomes showed a similar increase in in gene expressions of FAS and FABP4, while PC liposomes and DC-Chol/DOPE liposomes had no or only minor stimulating effect. As expected, the increase in lipid accumulation level is positively correlated with the gene expressions of FAS and FABP4 (Figure 6C,D). Overall, PC/DOTAP liposomes have more potential to induce adiposity in 7F2 osteoblasts than other liposomal formulations.

### 2.7. The Impact of Liposomal Formulations on Osteogenesis in 7F2 Osteoblasts

**Figure 7:**
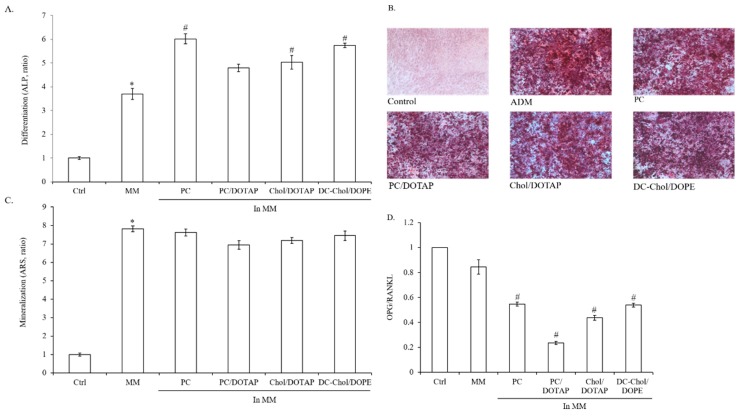
The impact of liposomal formulations on osteogenesis of 7F2 osteoblasts. 7F2 osteoblasts were treated with MM to induce osteoblast differentiation and mineralization. (**A**) The ALP activity of 7F2 osteoblasts in the absence and presence of MM with different liposomal formulations; (**B**) Histochemical staining of mineralization deposition. 7F2 osteoblasts were cultured in MM with different liposomal formulations for 14 days and calcium ion deposition was visualized by Alizarin Red S staining (×100 magnification, *n* = 3). Red staining represents mineral deposition. Photographs represent osteoblast cultures under bright field; (**C**) The quantification of osteoblast mineralization. Alizarin Red S was extracted with 10% cetylpyridinium chloride and quantified as described in Materials and methods; (**D**) The effect of liposomal formulations on the OPG/RANKL ratio of 7F2 osteoblasts. In order to determine gene expression of OPG and RANKL, 7F2 osteoblasts were incubated in MM with various liposomal formulations for 3 days and analyzed by real-time RT-PCR (relative to GADPH). The data are presented as the means ± S.E. * *p* < 0.01 relative to Control, ^#^ *p* < 0.01, relative to MM-treated cells.

In order to investigate the impact of liposomal formulations on osteogenesis, 7F2 cells were incubated in MM with various liposomal formulations for 10 days to determine ALP activities. In contrast to control, MM induced ALP activity around four times higher. Moreover, all the liposomal formulations can enhance ALP activities in 7F2 osteoblasts and that were 1.3 to 1.6 fold higher than ADM-treated cells (Figure 7A). Under MM condition, the calcium deposition level of 7F2 cells were around 8-fold higher than control (Figure 7B). Interestingly, the cells treated with liposomal formulations all exhibited similar calcium deposition level to MM-treated cells. Therefore, liposomal formulations have minor inhibitory effect on osteoblast mineralization no matter which types of lipids used for liposomal formulations (Figure 7C). Next, we evaluated the ratio of OPG/RANKL in cells incubated with various liposomal formulations. All the liposomal formulations reduced the ratio of OPG/RANKL but in different levels. PC and DC-Chol/DOPE liposomes demonstrated higher ratio of OPG/RANKL than other liposomes. Although there was no significant difference in calcium deposition levels in7F2 osteoblasts, the increase in ALP activity was positive correlated with the ratio of OPG/RANKL without considering the effect of MM. Among all the liposomal formulations, PC/DOTAP liposomes has the worst impact on osteogenesis of 7F2 cells (Figure 7D).

### 2.8. The Inflammatory Responses of IL-1β-stimulated Osteoblasts after Treatment with Various Liposomal Formulations

**Figure 8:**
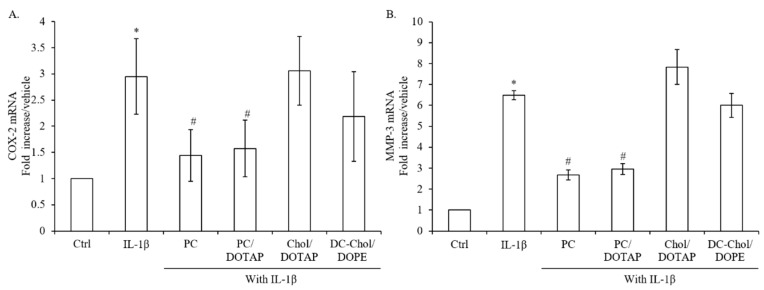
The effect of liposome formulations on the gene expression of pro-inflammatory mediators, COX-2 and MMP-3 in 7F2 osteoblasts. (**A**,**B**) Real-time RT-PCR analysis was performed in 7F2 osteoblasts after 4 h treatment of IL-1β with various liposome formulations. The data are presented as the means ± S.E. * *p* < 0.01 relative to Control, ^#^ *p* < 0.01 relative to IL-1β-stimulated cells.

In comparison with control, 7F2 osteoblasts showed around 3 and 6-fold higher gene expression of COX-2 and MMP-3 after 4 h of IL-1β treatment (Figure 8A,B). In addition, PC liposomes showed 51% and 59% reduction on IL-1β-induced expressions of COX-2 and MMP-3, while Chol/DOTAP and DC-Chol/DOPE liposomes had no or a minor inhibitory effect on COX-2 and MMP-3 expressions. Interestingly, PC/DOTAP liposomes showed the same inhibition pattern on IL-1β-stimulated inflammatory responses in 7F2 osteoblasts as that of PC liposomes. Therefore, PC-contained liposomes may perform the inhibitory potential on the inflammatory responses of 7F2 osteoblasts but not lipid alone.

## 3. Discussion

Recently, liposomal formulation become one of the interesting medications for osteoarthritis treatment because liposomal formulation can minimize the adverse effects and cytotoxicity of drugs and is able to carry hydrophilic and hydrophobic drugs on the same carriers. Oliveira et al. prepared small cationic liposomes such as DOTAP/DOPE or DOTAP/Chol liposomes in the mole ratio of 1:1 and that showed no significant cytotoxicity and good transfection activities in osteoblast-like cells, MG-63 and MC3T3-E1 cells [33]. Moreover, van der Geest et al. used long-circulating liposomes containing distearoyl-phosphatidylethanolamine-methyl-polyethylene glycol to encapsulate prednisolone to improve joint targeting and anti-inflammatory efficacy for the treatment of rheumatoid arthritis [34]. However, our previous study found that liposome treatment can slightly suppress osteoblast mineralization through the lipid accumulation [6]. Therefore, we prepared 4 different liposomal formulations at the same molar ratio but with different lipid compositions (PC, PC/DOTAP, Chol/DOTAP and DC-Chol/DOPE) to investigate the impact of lipid types and liposomal formulations on osteogenesis and lipid droplet formation in 7F2 osteoblasts. All the lipids used in this study are selected from liposome or lipid-based drug formulations which have been approved for human use [13,30]. Due to poor aqueous solubility, all the lipids were dissolved in ethanol for the cell culture studies. Ethanol is edible and have been used as the solvent for liposome preparation. By the way, the study reported by Kc et al. suggested that chronic alcohol exposure may increase susceptibility to the development and/or progression of OA [7]. The in vivo model of chronic alcohol treatment demonstrated that chronic alcohol consumption increased proteoglycan loss in both knee and shoulder joints of mice, and stimulated multiple inflammatory mediators involved in cartilage. Chen et al. also indicated that ethanol significantly increased lipid accumulation and up-regulated gene expressions of aP2 and PPARγ in mesenchymal stem cell line C3H10T1/2 [35]. Similar to previous studies, our results also showed that ethanol slightly stimulated intracellular lipid droplet formation and while suppressed calcium deposits in 7F2 osteoblasts but not in statistic difference. 

Importantly, Rezq et al. indicated that mice fed a diet containing olive oil, butter or animal fat had significant increase in bone density, while those fed diets containing soybean oil, corn oil, sunflower oil or margarine had significant decreases in femur bone density [36]. Therefore, the type of lipid has an important effect on bone health. The present study indicated that cationic lipids, DC-Chol, and DOTAP, could significantly increase lipid droplet formation and up-regulate the expressions of FAS and FABP4 in 7F2 osteoblasts and while suppress ALP activity and osteoblast mineralization. In addition, DC-Chol, and DOTAP could stimulate the gene expressions of COX-2 and MMP-3 in IL-1β-induced 7F2 osteoblasts and hence these cationic lipids can cause boss loss through the up-regulation of inflammatory responses. Except of cationic lipids (DC-Chol and DOTAP), Chol promoted more lipid droplet formation and FAS and FABP4 expressions than PC and DOPE. Our results are consistent with those of Soazig et al. who reported that Chol could induce lipid droplet formation in 3T3-L1 adipocytes [37]. Interestingly, Manickam et al. mentioned that eicosapentaenoic acid (EPA)-treated adipocytes, with reduced lipid droplet size and total lipid accumulation, have improved inflammatory response [38]. In the cellular model of endothelial inflammation, i.e., HMEC-1 cells exposed to TNF-α, Czamara et al. revealed that the formation of lipid droplets can be directly correlated with the increase production of prostacyclins-endogenous inflammation mediators [39]. Correspndingly, Asterholm et al. demonstrated that the degree of high fat diet-induced obesity could be reduced in a mouse model with dominant-negative TNF-α expression and hence obesity could be responsible for the induction of inflammatory responses [40]. Here, DC-Chol and DOTAP with increased lipid droplet formation, had higher inflammatory responses. Compared with cationic lipids (DC-Chol and DOTAP), neutral lipids (PC and DOPE) exhibited lower cytotoxicity, less inflammatory responses, reduced lipid accumulation, and lower inhibition of osteoblast differentiation and mineralization. Hence, Chol, DC-Chol and DOTAP may reduce bone mineral density through the increase of inflammatory responses and intracellular lipid droplet formation. Among the lipids, DC-Chol showed the most intensive induction of adipogenesis, while PC is considered to be the best lipid due to relatively minor effect on osteoblast differentiation and mineralization. Therefore, the behavior of osteoblasts under different lipid conditions can be arranged in the following rank order PC > DOPE > Chol > DOTAP > DC-Chol.

Similar to lipids, all the liposomal formulations stimulated lipid droplet formation but the level of lipid droplet formation was different. Among these liposomal formulations, DOTAP containing liposomes (PC/DOTAP and Chol/DPTAP) stimulated more lipid droplet formation and adipogenic marker expressions than others. On the other hand, all the liposomal formulations trend to increase ALP activity and there is no significant adverse effect observed on osteoblast mineralization. Although DC-chol induced high lipid droplet formation and intensive expressions of FAS and FABP4, incorporation of DC-chol into liposomes (DC-chol/DOPE liposomes) showed a similar osteoblast behavior to PC liposomes. Therefore, incorporation of DC-chol or Chol into liposomes may have a minor effect on osteoblast differentiation. Interestingly, PC containing liposomes (PC only and PC/DOTAP) suppressed IL-1β-induced gene expression of COX-2 and MMP-3 but not Chol/DOTAP liposomes or DC-Chol/DOPE liposomes. It is worth to note that PC liposomes were the only neutral liposomes in four formulations and PC/DOTAP and DC-chol/DOPE liposomes had similar surface charges. However, PC/DOTAP and DC-CHol/DOPE liposomes showed different characteristic features in adipogenic differentiation, osteogenesis and anti-inflammation. Therefore, liposomal charge and lipid composition both play important roles in 7F2 osteoblast differentiation and inflammation. In consistent with our previous studies, we found PC containing liposomes show good anti-inflammatory activities [6]. In the human intestinal epithelial cell line Caco-2, PC down-regulated TNF-α-induced gene expressions of pro-inflammatory cytokines such as ICAM-1, MCP-1, IL-8 and IP-10 through the inhibition of NFκB activation and hence lipid based therapy with PC may have benefits for ulcerative colitis, through anti-inflammatory effect [41,42]. Taken together, the behavior of osteoblasts under the stimuli of different liposomal formulations can be arranged in the following rank order PC liposomes > DC-Chol/DOPE liposomes > PC/DOTAP liposomes = Chol/DOTAP liposomes.

It is important to identify how lipid types and liposomal formulations can affect osteoblast behaviors such as cell viability, differentiation, mineralization and inflammatory responses. Similar to other researches, our results also demonstrated a reciprocal and inverse relationship between lipid droplet formation and osteoblast mineralization in 7F2 osteoblasts [1]. Hence, lipids or liposomal formulations may reduce bone mineral density through the increase of inflammatory responses and intracellular lipid droplet formation. As mentioned previously, OA patients treated with Meriva, a curcumin-PC complex, showed significant reduction in pain, stiffness and physical functions in comparison with the patients who were managed using the best available treatment. Furthermore, OA patients treated with Meriva^®^ had reduced levels of inflammatory markers (IL-1β, IL-6, sVCAM-1 and ESR) in blood [8]. Though the increase of drug bioavailability, Ibrahim et al. demonstrated that Meriva suppressed the expression of MMP-9 and lung metastasis more effectively than free curcumin in the animal model of mammary gland adenocarcinoma [43]. Therefore, PC containing liposomes might exert a potent therapeutic efficacy in bone diseases.

## 4. Materials and Methods

### 4.1. Materials and Cell Culture

Phospholipon 90G (phosphatidylcholine 90%) is purified PC from soybean lecithin provided by American Lecithin Company (Oxford, CT, USA). DOPE, DC-Chol, and DOTAP were acquired from Avanti (Alabaster, AL, USA). Oil Red O Staining kit was obtained from Lifeline (Frederick, MD, USA). Chol and Alkaline Phosphatase Colorimetric Assay kit were purchased from Sigma-Aldrich (St Louis, MO, USA). All cell culture materials including Dulbecco's modified eagle's medium (DMEM), fetal bovine serum (FBS), l-glutamine, adipocyte differentiation medium (ADM) were purchased from Gibco (Grand Island, NY, USA). All solvents used for analytical grade are obtained from J.T. Baker (Center Valley, PA, USA), and cytokines were purchased from ProSpec (Saint Louis, MO, USA). Mouse osteoblast-like cells (7F2) were acquired from food industry research and development institute, Taiwan. Cell lines 7F2 were cultured in DMEM supplemented with 10% *v*/*v* FBS, 100 units/mL penicillin and 100 μg/mL of streptomycin. Cells were maintained at 37 °C with 5% CO_2_ in a humidified incubator.

### 4.2. MTT Cell Growth Assay

7F2 osteoblasts were seeded at a density of 10^4^ cells/well in 96-well plates to determine cell viability. Culture media were replaced with the media containing test samples (lipids or liposomal formulations) at various concentrations. After 1 day of incubation at 37 °C in a 5% CO_2_ atmosphere, all cell supernatants were removed and 200 μL of 3-(4,5-dimethylthiazol-2-yl)-2,5-diphenyltetrazolium bromide (MTT) reagent (100 μg/mL) was added to each well for another 4 h incubation. 100 µL DMSO was added to each well to dissolve formazan crystals and the absorbance was measured at 570 nm using the ELISA reader (Infinite M200, Tecan, Männedorf, Switzerland). All experiments were performed in quadruplicate, and the relative cell growth (%) was expressed as a percentage relative to the untreated control cells.

### 4.3. Liposomal Formulation

Liposomes with different compositions (PC only, PC/DOTAP, Chol/DOTAP, and DC-Chol/DOPE at 1:1 molar ratio) were prepared by the modified thin-film hydration method [6]. Different lipids were dissolved in the mixture of ethanol and chloroform. After lipids were completely dissolved in a round-bottom flask, the solvent was evaporated by rotary evaporator (Eyela, N-1000, Tokyo, Japan) at 45 °C and vacuum dried to form a dry lipid film. The lipid film was then hydrated with 2 mL PBS. The hydrated solution was downsized by sequence passing through 400 nm and 100 nm polycarbonate membranes using an extruder (Avanti Mini Extruder, Alabaster, AL, USA) to obtain uniformly-sized liposomes.

### 4.4. Particle Characterization

The particle size distribution of liposomes was determined using a dynamic light scattering instrument (LB-550, Horiba Ltd., Kyoto, Japan). Liposomal dispersions were diluted with double-distilled water to ensure the light scattering intensity in the instrument’s sensitivity range. The stability of the liposomal formulations was evaluated after storage at 4 °C for 1, 7 and 14 days. All measurements were taken in triplicate.

### 4.5. Oil Red O Staining

7F2 osteoblasts were cultured at a density of 5 × 10^4^ cells/well in 6-well plates in DMEM containing 10% FBS and 10% ADM with different types of lipids or liposomal formulations at 37 °C in a 5% CO_2_ atmosphere. After 7 days of incubation, the supernatants were removed and cells were washed with PBS and fixed with 1 mL of 4% paraformaldehyde fixative solution for 20 min at room temperature. After the reaction, cells were rinsed with PBS and treated with 1 mL of 1,2-propanediol dehydration solution for 5 min. Then, 1 mL of Oil Red O stain solution was added and incubated at 37 °C for 30 min of adipocyte staining. Later, cells were treated with 1 mL of 1,2-propanediol stain differential solution for adipogenic differentiation. The images of cell morphology were taken by microscope (Eclipse Ti-E, Nikon Corporation, Tokyo, Japan) and CCD camera system (SPOT RT3, Diagnostic instruments Inc., Sterling Heights, MI, USA). Finally, 1 mL of isopropanol was added to each well and shaken for 10 min to dissolve the dye, then the absorbance was measured by an ELISA reader at a wavelength of 520 nm. Each experiment was taken in triplicate.

### 4.6. Alkaline Phosphatase (ALP) Activity Analysis

7F2 cells were seeded at a density of 10^4^ cells/well in 24-well plates. Mineralization medium (DMEM containing 10% FBS, 5 mM β-glycerophosphate, and 50 μg/mL ascorbic acid) with different types of lipids or liposomal formulations was added to each well and incubated for 10 days at 37 °C in a 5% CO_2_ atmosphere. The supernatants were removed and washed by PBS. Cells were lysed with 150 µL of assay buffer, shaken for 10 min, and centrifuged at 12,000× *g* for 10 min at 4 °C. After centrifugation, the supernatant was collected and transferred to a clean tube. 2–80 µL of the testing samples was added to each well and the volume was adjusted to 80 µL/well with assay buffer. 50 µL of 5 mM *p*-nitrophenyl phosphate (pNPP) solution was added to each testing sample and background control. The samples were well mixed and incubated in the dark condition at 25 °C for 60 min. Alkaline phosphatase will convert the substrate, pNPP, to an equal amount of colored *p*-nitrophenol (pNP). The absorbance was measured by an ELISA reader at a wavelength of 405 nm. The measurements were taken in triplicate.

### 4.7. Alizarin red S Staining for Osteoblast Mineralization

7F2 osteoblasts were seeded at a density of 10^4^ cells/well in 24-well and cultured in 2 mL mineralization medium (MM) with different types of lipids or liposomal formulations for 14 days. After incubation, the samples were washed with PBS, fixed with 75% *v*/*v* ethanol and dried at room temperature. After complete drying, the fixed cells were stained with 200 μL of 1% Alizarin Red S for an hour. Cell morphology was imaged by microscope (Nikon TI-E) and CCD camera system (SPOT RT3). 400 μL of 10% *w*/*v* cetylpyridinium chloride solution was added to each well and shaken for 10 min to dissolve calcium. Finally, the absorbance was measured by an ELISA reader at a wavelength of 560 nm. The measurements were taken in triplicate.

### 4.8. Quantitative Real-Time PCR

7F2 osteoblasts were seeded in 6 cm dishes for 24 h of incubation. Then, 7F2 cells were incubated with ADM for 7 days to induce adipogenesis or with MM for osteoblast differentiation. For inflammation evaluation, 7F2 osteoblasts were stimulated with IL-1β (10 ng/mL) to induce inflammatory responses. After treatment, total RNA was extracted using the Trizol reagent (Protech Technology, Taipei, Taiwan) following the protocol to the manufacturer’s instructions. RNA (2 μg) was reverse transcribed using TProfessional Basic (Biometra GmbH, Göttingen, Germany). The cDNA (equivalent to 20 ng) was used in an StepOnePlus™ Real-Time PCR System using FastStart DNA Master-PLUS SYBR Green I (Applied Biosystems, Foster City, CA, USA). The designed primers were shown in Table 2 and all primers using nucleotide sequences present in the PrimerBank database. Each sample was corrected using the mean cycle threshold (CT) value for GAPDH. Relative gene expression was analyzed using the ΔCT method and expressed as fold change (2^−ΔCCT^) T relative to the expression values in non-stimulated cells.

### 4.9. Statistical Analysis

The experiments were repeated at least twice with similar results, and the values were expressed as means ± standard deviations. The data were analyzed by the Dunn’s post-test using SPSS Version 12 (IBN, New York, NY, USA) and Sigma Plot (San Jose, CA, USA). Differences were considered to be statistically significant at *p* < 0.05.

## Figures and Tables

**Table 1:** Physical parameters of the liposomal formulations after extrusion.

Liposome Type	PC	PC/DOTAP	Chol/DOTAP	DC-Chol/DOPE
Particle size (nm)	174.9 ± 4.7	162.4 ± 3.5	164.3 ± 4.0	174.8 ± 6.0
PDI	0.056 ± 0.019	0.133 ± 0.029	0.093 ± 0.025	0.122 ± 0.020
Zeta Potential	−5.09 ± 1.3	+37.4 ± 2.3	+50.9 ± 2.7	+34.2 ± 2.5

**Table 2:** Sequences of primers used in real-time PCR experiment.

Target	Forward (5′~3′)	Reverse (5′~3′)
GAPDH	CATGAGAAGTATGACAACAGCCT	AGTCCTTCCACGATACCAAACT
FAS	CCACTGAAGAGCCTGGAAGA	GTAGTCAGCACCCAAGTCCT
FABP4	AGTGAAAACTTCGATGATTACATGAA	GCCTGCCACTTTCCTTGTG
OPG	CCTTGCCCTGACCACTCTTAT	CACACACTCGGTTGTGGGT
RANKL	CGCTCTGTTCCTGTACTTTCG	GAGTCCTGCAAATCTGCGTT
COX-2	CAGCCAGGCAGCAAATCC	ACATTCCCCACGGTTTTGAC
MMp-3	GGCCTGGAACAGTCTTGGC	TGTCCATCGTTCATCATCGTCA
